# The Doctor in the Witness Box

**Published:** 1856-07

**Authors:** 


"*The Doctor in the Witness Box.*"—Such is the title of an article in the "Dublin University Magazine," based on the medical evidence of the "Great Burdon slow poisoning case." Its *gravamen* is an attack on medical evidence, generally, and its value in criminal investigations; and we are sorry to say, that in this individual case the reasoning of the critic is too well supported. But we would not reason from this case alone, neither would we adopt the conclusions of the article. We have the highest confidence in medical evidence when it is intelligent, while, at the same time, we are duly conscious of the many shortcomings to which it is peculiarly liable.

But the "Great Burdon" case furnishes a text from which one may reason, and we are willing to accept it as a proper one.

Jane Wooler, wife of Joseph Smith Wooler, of Great Burdon, in the county of Durham, England, died in June last, with symptoms which might be due to chronic gastro-enteric inflammation, or to the gradual administration of arsenic in small doses. She was sick for some weeks, during which time she was under the care of three medical gentlemen. The physicians seem to have arrived separately at the suspicion of poisoning, from the fact that they were aware of some Fowler's solution of arsenic which was in the family medicine chest. Although this suspicion was early excited, they do not seem to have taken any measures to prevent the administration of the poison, or to furnish antidotes for it, contenting themselves with dark hints to each other, with analyzing the secretions, and with calling for a coroner's inquest after the death of the lady.

Previously to the inquest they held a post-mortem examination, and removed some of the viscera for the purpose of analysis.

We content ourselves with stating in general terms that the symptoms before death were those of chronic gastro-enteric inflammation, whether idiopathic or arsenical, and that the *post-mortem* appearances corresponded to them; that Mr. Henzell, the assistant of one of the practitioners in attendance, thought that he discovered arsenic in the secretions before death; that Prof Christison, of Edinburgh, did the same; while Mr. Richardson, (chemist,) of Newcastle, and Prof. Taylor, the celebrated medico-jurist, of London, each detected very small quantities of arsenic, but in very minute quantity, proven.

But the question arises now, were these medical men justified in placing upon these facts the interpretation they did, viz: that the arsenic was *feloniously* administered, and that Mr. Wooler was the criminal?

In the first place no motive was proven. Mr. Wooler was an affectionate hnsband, kind and attentive to his wife during her illness. Her sick room was filled with interested people. Her relatives were brought together by Mr. Wooler, and were constantly about her. No overt act on his part, and no motive for any were proven. When the suspicion of poison first came to his ears, on the day of his wife's death, he requested her sister to take personal charge of the medicine chest, so that nothing might be removed. Not dreaming that he was himself the victim of the inquisition, he requested the *post-mortem* examination, and paid Dr. Taylor for his chemical analysis. In fact there was no reason for suspecting Mr. Wooler except that he had the opportunity to poison his wife if he chose; but, for that matter, so had the doctors, and to our mind the suspicion of poisoning might as well attach to them as him.

But if we except Mr. Wooler from the list of those liable to suspicion, the question arises, who did poison her? Or was she poisoned at all?

To solve this problem we must look at the quantity of arsenic found. Mr. Richardson, Profs. Christison and Taylor, between them, found less than a grain of arsenic from an analysis of the heart, lungs, stomach, liver and intestines. We will not question the analysis—it was, doubtless, as accurate as any test for arsenic can be, and will grant that some one had given her arsenic — whether in poisonous doses or not we do not say, though the fact that she had tuberculosis might very well account for her death.

But who gave her the arsenic? We verily believe the doctors did it, though not feloniously. Among the various medicaments given by them to the patient, we find the following truly English list: *Nitrate of silver*, strychnia, extract of lettuce, quinine, *sulphate of copper*, opium, nux-vomica, henbane, *acetate of lead*, strong acetic acid, morphine, *blue pill*, *iodide of potash*, *bismuth*, and *nitro-muriatic acid*. Now, how much of this *olla podrida* of physic is liable to adulteration with arsenic? And how far would an alloy of the metallic salts in the list go to confuse the arsenical tests?

No man knows, and no man ought to have sworn that a suspicion of poison could properly be based on such evidence. Again we should state that the patient had lived in the East Indies, and had, for aught we know, taken Fowler's solution there.

So much for Mrs. Wooler's case, which our readers may find in "Littell's Living Age" for April 12, 1856.

But does this instance of perverted scientific acumen impair the true value of medical evidence? On the contrary we regard it as proving that chemistry is one of the most exact and reliable means for the detection of crime. Here these chemists search a body in which it was evident only very small quantities of arsenic existed, and drag from its hiding place in the tissues the single grain which proved it. If they gave their evidence in a true and single spirit, they deserved the warmest encomiums for their skill. But they seem to have gone beyond this, and lent themselves as a new kind of detective police, and to have hunted down poor harmless Mr. Wooler, assuming in the outset, not only that there had been a crime, but that he was the criminal!

Here was their fault. Had they confined themselves to their proper duty — the detection of any poison that may have existed — or had the attending physicians paid as much attention to prevention as they did to detection, Mrs. Wooler might have been still alive, or, if dead, an innocent man would have been spared a disgraceful trial, and the medical profession would have escaped the scathing rebuke administered under the title of the "Doctor in the Witness Box."

It is no part of the business of the physician or the toxicologist to become a detective policeman. His duty in criminal jurisprudence is simply that of the intelligent citizen. If by his superior learning he is able to recognize facts as criminal which would escape the common eye, his duty to society requires that he should make them known. If he is required to testify in cases of poisoning, he should confine himself to the question of the existence of poison in the tissues, leaving to the proper authorities the task of discovering who put it there. In autopsies made for purposes of jurisprudence, he should not lend himself to a persecution of the prisoner. His duty is to seek out the cause of death. If he finds the skull fractured, let him say so. Perhaps it was done by accident, perhaps by the hand of another, but that is none of his business—let him leave that question to the jury. Above all, let him avoid any appearance of a desire to convict the prisoner. If his evidence, given in a fair, unbiassed way, tends toward conviction, he should regard it as a painful but imperative duty; but it seems to us that in all cases where two constructions, one leaning toward mercy, the other toward punishment, may be put on any fact, his duty is to state them both fairly.

Doctors are usually witnesses for the prosecution, but they should never consider themselves as a part of the prosecution. Their business is to save life, not to destroy it; to repair the ravages of crime, not to bend themselves to its detection.

